# Neurological complications after thoracic endovascular aortic repair. Does the left subclavian artery coverage without revascularization increase the risk of neurological complications in patients after thoracic endovascular aortic repair?

**DOI:** 10.1186/s13019-018-0825-3

**Published:** 2019-01-08

**Authors:** Dariusz Janczak, Agnieszka Ziomek, Jakub Kobecki, Maciej Malinowski, Kornel Pormańczuk, Mariusz Chabowski

**Affiliations:** 1 Department of Vascular, General and Transplantation Surgery, Faculty of Postgraduate Medical Training, Wroclaw Medical University, 213 Borowska street, 50-556, Wroclaw, Poland; 2 Department of Surgery, 4th Military Teaching Hospital, 5 Weigla street, 50-981, Wroclaw, Poland; 3 Division of Surgical Specialties, Department of Clinical Nursing, Faculty of Health Science, Wroclaw Medical University, 5 Bartla Street, 51-618, Wroclaw, Poland

**Keywords:** Thoracic endovascular aortic repair (TEVAR), Left subclavian artery (LSA), Cerebrovascular accident (CVA), Stroke, Spinal cord ischemia (SCI)

## Abstract

**Introduction:**

One of the most severe complications after TEVAR is ischemic stroke and spinal cord ischemia (SCI) resulting in severe disability. These complications can be fatal up to 30% of cases, so it is very important to define risk factors associated with the occurrence of such events. The aim of this study was to define the causes and risk factors associated with the occurrence of neurological complications in patients after TEVAR.

**Materials and methods:**

We performed a retrospective analysis of 51 patients undergoing TEVAR in the Department of Vascular Surgery of Military Teaching Hospital in Wroclaw between 2014 and 2017. In 18 patients LSA coverage was managed without revascularization (35.29%), and in 33 patients LSA remained uncovered (64.71%).

**Results:**

We did not find any statistically significant difference in the incidence of stroke and spinal cord ischemia in patients with covered and uncovered LSA (stroke *p* = 0.37, SCI *p* = 0.58). In the group of patients with covered and uncovered LSA, we did not find any significant differences in the incidence of additional comorbidities such as obesity, ischemic heart disease, hypertension or previous stroke.

**Conclusions:**

There is no difference in stroke and SCI occurrence between patients with covered and uncovered LSA. Although there are many studies analysing the risk of such complications, there is no specific consensus regarding the treatment of LSA coverage. Randomised clinical trials on a large group of patients are still needed.

## Introduction

Over the last 20 years, the treatment of thoracic aortic conditions has been completely revolutionized by introducing thoracic endovascular aortic repair (TEVAR). Endovascular treatment is associated with reduced mortality and shorter hospital stay compared to open repair [1, 2]. Moreover, such treatment is also more widely accepted by the patients themselves. One of the most severe complications of TEVAR is ischemic stroke and spinal cord ischemia (SCI). The cause of spinal cord ischemia and lower limb paresis is wide coverage of the aorta wall with stentgraft, which closes the arteries that are supplying blood to the spinal cord. Although the risk of SCI and stroke is considered to be lower than in open surgery, it is still as high as 2.5–5% [3]. Neurological complications can be fatal in up to 30% and are associated with long-term disability and handicap, which generates high costs. Therefore, it is very important to define the risk factors associated with such complications in order to prevent them as effectively as possible. Although there is no strong evidence on this subject, it is believed that one of the main risk factors for neurological complications is coverage of the left subclavian artery without revascularization. Some papers argue the opposite, that covering LSA without revascularization does not increase the incidence of neurological complications.

The aim of our work was to define the causes and risk factors associated with neurological complications in patients after TEVAR.

## Materials and methods

The records of 51 patients undergoing TEVAR between 2014 and 2017 were retrospectively analysed. Landing zone 0 and 1 was an exclusion criterion. Ischemic stroke was defined as any new loss of neurological functions of vascular ethiology lasting more than 24 h. TIA was defined as any new loss of neurological function of vascular ethiology lasting less than 24 h. Spinal cord ischemia (SCI) was defined as a permanent reduction or complete loss of neurological functions in the limbs. Each patient had thoracic and abdominal angiography before and three months after the surgery. Demographics and comorbidities of the patients are reported in Table 1. The mean age of the patient was 66.37 years. Most of the patients were male (*n* = 34, 66.67%). 38 patients (74.58%) were hypertensive, 34 (66.67%) patients had a BMI above 25, and almost half of the population was burdened with tobacco use (*n* = 24, 47.05%). Additionally in 13 cases (25.49%) thoracic aneurysm coexisted with an abdominal aneurysm.

**Table 1:** Demographics and comorbidities of the studied population

Variable	Number	%
Number of patients	51	
Female sex	17	33.3
Age,years	66.3	
BMI < 25	35	68.6
BMI 25–30	5	9.8
BMI 30–35	11	21.5
Urgent admission	34	66.6
TEVAR due to aneurysm	25	49
TEVAR due to acute type B dissection	26	51
Average diameter of aneurysms (cm)	8.39	
Coexistence of AAA	13	25.4
Tobacco use	24	47
Hypertension	38	74.5
Coronary artery disease	13	25.4
Antiplatelet therapy	15	29.4
Statin therapy	17	33.3
Previous stroke	6	11.7
Asymptomatic carotid stenosis	2	3.9

The indication for TEVAR was a thoracic aortic aneurysm (*n* = 25, 49%) or acute type B aortic dissection (*n* = 26, 51%). Two patients were operated due to thoracic aortic aneurysm rupture. The vast majority of patients were admitted and operated in urgent mode (*n* = 34, 66.67%). The main symptom in patients undergoing TEVAR was chest pain (*n* = 22, 64.7%). The strategy for managing TEVAR with landing zone 2 was to leave the left subclavian artery covered without revascularization. Patients were qualified for each type of procedure (i.e. covered or not covered LSA) due to the aneurism’s anatomy. When the aneurysm included the aorta directly behind the subclavian artery, there was a need to cover the subclavian artery in order to get a place to attach the stentgraft. In 18 cases LSA was covered without revascularization (35.29%), and 33 remained uncovered (64.71%). The average procedure time was 80.1 min and the average hospital stay was 7.59 days.

### Statistical analysis

The analysis of collected data was carried out using TIBCO StatSoft Statistica™ version 13.2 on a computer with the Windows operating system. T-student test and U-Mann-Whitney test were used to compare quantitative data such as mean values, standard deviations or medians. The chi-square test was used to analyse nominal variables, using Yates correction in some cases. The test results for *p* < 0.05 were considered statistically significant.

## Results

In the study population we found no statistically significant differences in the incidence of additional comorbidities such as obesity, ischemic heart disease, hypertension or previous stroke regarding LSA coverage (covered or not covered) (Table 2). In a 30-day observation, 4 patients (7.84%) experienced ischemic stroke. One patient, who experienced stroke, suffered from carotid artery stenosis. In one patient, the stroke was fatal. The mean age of the patient suffering stroke was 86.57 years. In 3 patients (5.88%) spinal cord ischemia in the form of lower limb paresis occurred after TEVAR. In one of these patients, a number of complications led to his death. Other complications include: reoperation due to hematoma or pseudoaneurysm in the groin (*n* = 6, 11.7%), arm ischemia requiring surgical intervention (*n* = 1, 1.96%), wound suppuration (*n* = 2, 3.92%), delirium (n = 2, 3.92%). Complications are collected in Table 3. In the study population we did not find a statistically significant difference in the incidence of stroke and spinal cord ischemia in patients with covered and uncovered LSA (stroke *p* = 0.37, SCI *p* = 0.58). Four out of 33 patients experienced stroke in LSA non covered group and no patient experienced stroke in LSA covered group. When it comes to SCI one out of 18 patients experienced SCI in LSA covered group and 2 out of 33 patients in LSA non-covered group. Although the incidence of neurological complications appears to be greater in non – covered LSA group, it does not reach statistical significance due to the small number of the patients. Number of complications such as myocardial infarction (*p* = 0.53), or death (*p* = 0.96) was also not significantly different (Table 4). Patients with neurological complications did not differ in additional comorbidities from patients without neurological complications (Table 5**).**

**Table 2:** Characteristics and comorbidities in patients with covered and non – covered LSA

Feature	LSA covered	LSA non -covered	P	
	18	33	51	
%	35.2%	64.7%		
BMI < 25	4	8		
%	22.2%	24.2%		
BMI25–30	13	21	*p* = 0.7826	
%	72.2%	63.6%		
BMI > 30	1	4	*p* = 0.8458	
%	5.5%	12.1%		
Hypertension	12	26	*p* = 0.7138	
%	66.6%	78.7%		
Previous stroke	0	6	*p* = 0.1953	
	0.0%	18.1%		
Coronary artery disease	2	11	*p* = 0.3667	
%	11.1%	33.3%		
Urgent admission	15	19	*p* = 0.4135	
%	83.3%	57.5%		
Acute type B dissection	12	14	*p* = 0.3554	
%	66.6%	42.4%		
Aortic aneurism	6	19	*p* = 0.3197	
%	33.3%	57.5%		
Male sex	11	23	*p* = 0.7793	
%	61.1%	69.7%		
Antiplatelet therapy	4	11	*p* = 0.7554	
%	22.2%	33.3%		
Statin therapy	7	10	*p* = 0.6631	
%	38.8%	30.3%		
			uMW	t-student
Mean age,years	64.6	67.3	*p* = 0.4838	0.47439
Hospitalization time	7.8	7.4	*p* = 0.3104	0.7758

**Table 3:** Complications after TEVAR

Complications after TEVAR	Number	%
Ischemic stroke	4	7.8
Spinal cord ischemia	3	5.8
Acute renal failure	2	3.9
Myocardial infarction	3	5.8
Death	4	7.8
Arm ischemia	1	1.9
Pseudoaneurysm or hematoma in the groin	6	11.7
Wound suppuration	2	3.9
Delirium	2	3.9

**Table 4:** Univariate analysis of post TEVAR complications in patient with covered and non – covered LSA

Feature	LSA covered	LSA non- covered	P
Stroke	0	4	*p* = 0.3706
%	0%	12.1%	
SCI	1	2	*p* = 0.5803
%	5.5%	6%	
ARF	1	1	*p* = 0.7443
%	5.5%	3%	
Cardiac arrest	1	1	*p* = 0.7443
%	5.5%	3%	
Death	2	2	*p* = 0.9609
%	11.1%	6%	
MI	0	3	*p* = 0.5286
%	0%	9%	
Neurological complications in total	1	6	*p* = 0.4958
	5.5%	18.1%

**Table 5:** Univariate analysis of characteristics and comorbidities in patients with and without neurological complications

Feature		Neurological complication	Without neurological complication			
AGE	<=70	3	27	30	*p* = 0.6944	
	> 70	4	17	21		
BMI	< 25	7	29	36		
	25–30	7	27	34		
	> 30	0	15	15		
Hypertension	yes	5	33	38	*p* = 0.7751	
	no	2	11	13		
Previous stroke	yes	1	5	6	*p* = 0.6696	
	no	6	39	45		
Coronary artery disease	yes	1	12	13	*p* = 0.8582	
	no	6	32	38		
Urgent admission	yes	4	30	34	*p* = 0.9417	
	no	3	14	17		
Aortic aneurism	–	1	24	25	*p* = 0.1843	
Acute type B dissection	–	6	20	26	UMW	t-student
Mean age, years		70.2	65.7		0.4353	0.3901
Average hospital stay		10.1	7.8		0.2982	0.1043

## Discussion

LSA coverage is necessary to obtain a landing zone in almost one third of the patients qualified for TEVAR [3]. The managing of the LSA (to revascularize or not) is not fully established. Some surgeons routinely perform LSA revascularization. Others selectively, yet another perform LSA revascularization only after symptoms appearance.

Whether the LSA coverage is associated with an increased or reduced risk of stroke remains unclear. So far, many studies have been conducted to compare the occurrence of neurological complications after TEVAR in relation to the management of LSA [4–7]. Most of these studies are retrospective ones, low in the strength of scientific evidence, and not randomized clinical trials.

In the meta-analysis taking into account researches from 1990 to 2008, it was found that the LSA coverage is associated with an increased risk of arm ischaemia and CNS ischemia in the vertebral - basilar circulation [4]. However, this was not associated with an increase risk of spinal cord ischaemia. In this study, no correlation was found between LSA coverage and higher risk of death, myocardial infarction or TIA. Evidence for this, however, is also weak, based mainly on retrospective studies.

The authors of the 2014 paper, who analysed 1010 patients after TEVAR in 2002–2010 also have similar views. They found that patients with LSA coverage are more likely to have ischemic stroke (*p* < 001), especially in posterior territory, and this is an important risk factor in patients undergoing TEVAR [5].

In a subsequent study of 1189 patients, the LSA coverage was found not to be associated with an increased risk of SCI or CVA [6]. There was no difference in SCI and CVA rates among patients undergoing LSA coverage with or without revascularization (SCI, 6.3% vs 6.1%, CVA, 6.7% vs 6.1%).

The authors of this work are opposed to the SVS recommendations. In 2009 r. SVS based on the analysis of 51 studies found that routine LSA revascularization is associated with a reduced number of neurological complications (stroke and spinal cord ischemia) [7]. Therefore, according to the guidelines, the Grade 2 Level C recommendation is to routinely revascularize LSA. Evidence for this are derived from mainly retrospective studies with low-quality scientific evidence rather than randomized clinical trials. In our study, we did not find a statistically significant difference in the incidence of neurological complications in patients with LSA coverage compared to patients with an uncovered LSA. However, the limitation of this study is the fact that a small group was analysed (*n* = 51).

It is now believed that the cause of stroke and SCI after TEVAR is multifactorial. One of the important elements causing the stroke is intraoperative embolism from the atherosclerotic plaques of the aortic arch, another reduction in the flow through the vertebral arteries [3, 8]. The reason for SCI is the covering of the intercostal arteries (mainly Th 9–12), Adamkiewicz’s artery, LSA and previous abdominal aortic aneurysm surgery [9–11].

Although TEVAR is associated with a reduced risk of neurological complications comparing to an open surgery, the risk is still high. In the recommendations for the descending aortic pathology issued by ESVS, the Class II level C recommendation is revascularization of covered LSA, but only in selected patients with an increased risk of neurological complications [3]. The authors state that this should be done in the case of the dominant left subclavian artery, after the left internal thoracic artery bypass, in patients with a large stent graft area (> 200 mm) and in patients after an abdominal aortic aneurysm surgery (earlier covering or ligation of the lumbar arteries). In our study population, no patient met such criterion, therefore all the patients with LSA covered were left without revascularization.

When deciding on LSA revascularization, one should also remember about complications that may occur, such as: cardiovascular accident, injury of brachial plexus, subclavian vein, vagus nerve, diaphragmatic nerve or thoracic duct. It is said that up to 4.4% of patients suffer from diaphragmatic nerve damage [4].

What can we do to reduce the risk of neurological complications? A recently published study analysing the results of TEVAR showed twice as frequent occurrence of ischemic stroke in patients with thoracic aortic aneurysm, compared to dissection [2]. This may be associated with more frequent aortic arch atheroma, vulnerable to cause thromboembolism in patients with aneurysm. Atherosclerotic disease of the aortic arch is also known factor for neurological events after open heart surgery or carotid stenting. This gives grounds for conjecture that perhaps high-dose statin therapy resulting in stabilization of the atherosclerotic plaque could reduce the risk of perioperative strokes after TEVAR [2].

The way to reduce SCI is cerebrospinal fluid drainage. It is still unclear when to introduce such a treatment. According to ESVS, drainage of cerebrospinal fluid should be used in patients who have a large aortic area covered by the stent graft (> 200 mm), or with previous abdominal aortic aneurysm surgery, because the benefits of drainage are best recognised in patients at high risk of spinal cord injury [3]. In our patients, fluid drainage was performed in patients with a large aortic area stent coverage.

## Conclusions

In our study, we did not find statistically significant differences in the incidence of neurological complications in patients with covered and uncovered LSA. Although there are many studies analysing the risk of such complications, there is no specific consensus regarding the treatment of LSA-covered. Randomized clinical trials on a large group of patients are still needed.

## Abbreviations

BMI: Body mass index
CNS: Central nervous system
CVA: Cerebrovascular accident
ESVS: European Society for Vascular Surgery
LSA: Left subclavian artery
SCI: Spinal cord ischemia
TEVAR: Thoracic endovascular aortic repair
TIA: Transient ischemic attack
TIBCO: The Information Bus Company
